# Severe mitral stenosis as a cause of paradoxical low-flow, low-gradient severe aortic stenosis: an explorative study on hemodynamics and outcomes

**DOI:** 10.3389/fcvm.2025.1634914

**Published:** 2025-10-31

**Authors:** Dania Mohty, Mohamed H. Omer, Josef Marek, Omar Ahmad, Waleed Alhemayed, Mohammed F. Janjua, Romain Capoulade, Mohammed Alhumaid, Khadija Alassas, Hani Sergani, Philippe Pibarot, Bahaa M. Fadel

**Affiliations:** 1 Heart Center, King Faisal Specialist Hospital and Research Center, Riyadh, Saudi Arabia; 2 College of Medicine, Al Faisal University, Riyadh, Saudi Arabia; 3 School of Medicine, Cardiff University, Cardiff, United Kingdom; 4 Department of Cardiovascular Medicine, Faculty of Medicine, Charles University and General University Hospital in Prague, Prague, Czechia; 5 l'institut du Thorax, INSERM, CNRS, UNIV Nantes, Nantes, France; 6 Mohammed Bin Khalifa Bin Salman Al Khalifa Specialist Cardiac Centre, Manama, Bahrain; 7 Institut Universitaire de Cardiologie et de Pneumologie, Université Laval, Québec City, QC, Canada

**Keywords:** aortic stenosis, mitral stenosis, multiple valve disease, low-flow low-gradient aortic stenosis, rheumatic heart disease

## Abstract

**Background:**

Aortic stenosis (AS) and mitral stenosis (MS) are valvular heart diseases that may present concomitantly, particularly in regions where rheumatic heart disease remains prevalent. While each condition has been extensively studied in isolation, there is limited data on the clinical characteristics of patients with combined AS-MS.

**Methods:**

We retrospectively identified patients with significant AS and concomitant significant MS from the echocardiography database between 2003 and 2018. Exclusion criteria included left ventricular ejection fraction <50%, other significant valvular lesions, prior cardiac surgery, and associated congenital heart disease. Patients with isolated AS were compared to patients with combined AS-MS.

**Results:**

Of 1470 patients with severe AS, a total of 353 patients were included: 41 with combined AS-MS and 312 with isolated AS. The prevalence of combined AS-MS was 11% among patients with significant AS. Compared to patients with isolated AS, patients with combined AS-MS were significantly younger (50 vs. 63 years, *p* < 0.001), had a lower prevalence of hypertension (44% vs. 64%, *P* = 0.017) and diabetes (22% v. 42%, *p* = 0.013), and a greater prevalence of atrial fibrillation (17% vs. 5%, *p* = 0.003). Patients with combined AS-MS had a significantly larger left atrial size (4.79 ± 0.70 cm vs. 3.93 ± 0.73 cm, *p* < 0.001), higher peak tricuspid velocities (3.14 ± 0.59 m/s vs. 2.72 ± 0.45 m/s, *p* < 0.001), and greater prevalence of moderate or severe tricuspid regurgitation (15% vs. 1%, *p* < 0.001). Echocardiographic parameters assessing transvalvular flow rate did not differ significantly between the two groups. After multivariate adjustment for age and gender combined AS-MS was associated with worse 5-year overall survival (HR 2.672, 95% CI 1.060–6.732, *p* = 0.037).

**Conclusion:**

Combined mitral and aortic stenosis is not uncommon (11%) but linked to worse outcomes than isolated AS. Despite expectations, concomitant significant MS did not increase prevalence of paradoxical low-flow, low-gradient AS.

## 1 Introduction

Multiple valvular heart disease (MVHD) is frequently observed among patients with rheumatic heart disease or in elderly patients with degenerative valve lesions (1). MVHD is a challenging diagnostic entity as a consequence of hemodynamic interactions that could hinder the accuracy of echocardiographic parameters which have only been validated in the setting of isolated valve disease (2). Moreover, MVHD has been associated with heightened morbidity and mortality and it is an area in which surgical valve interventions have been associated with increased futility when compared to isolated valve disease (3). The Euro Heart Survey of over 5,000 adults from 25 European countries identified a 20% prevalence of patients diagnosed with native poly valve lesions (4). The most frequent association found was the presence of concomitant aortic stenosis (AS) and mitral regurgitation for those with degenerative valve disease and aortic regurgitation and mitral stenosis for those with rheumatic valve disease. However, the prevalence and significance of combined mitral stenosis (MS) and AS is not well documented in the literature and remains largely unknown. The combination of AS and MS has been previously reported in 17% of 170 patients undergoing mitral and aortic surgery in Switzerland (5). More recently, Joseph et al. utilized a large U.S. transcatheter valve therapies registry to investigate the prevalence of MS in patients undergoing transcatheter aortic valve replacement (TAVR) (6). The study found that the prevalence of MS was 11.6% in this cohort, with 2.7% of patients having severe disease. Nonetheless, these findings emerge from developed countries wherein the most likely cause of MS is underpinned by degenerative valve disease and/or severe mitral annular calcification.

Coexistent AS and mitral stenosis is often poorly hemodynamically tolerated consequently resulting in the earlier onset of symptoms (7). MS in the setting of combined AS can significantly hinder preload and left ventricular filling, which are often already damaged as a consequence of concentric left ventricular hypertrophy, leading to a paradoxical low flow low gradient normal EF severe AS (7, 8). Indeed, an assumption exists that the pressure gradients across the aortic valve may be lower in the setting of associated AS + MS than that of isolated aortic valve disease for same stenosis severity. Consequently, the presence of concomitant significant MS is considered to be a cause of “paradoxical low flow low gradient” in patients with significant AS in the setting of preserved left ventricular ejection fraction (LVEF) (9). Furthermore, the presence of concomitant AS and MS impedes the evaluation of the severity of each lesion due to overlapping hemodynamic interactions between the two disorders (7, 9).

To our knowledge, there are no current reports highlighting the clinical features, hemodynamic, characteristics and outcomes of concomitant rheumatic MS and AS. In our region of Saudi Arabia and the Middle East, there is a heightened prevalence of rheumatic valve disease (10). Therefore, using a single tertiary center, where rheumatic valve disease remains the leading cause of valvular disease, our study aimed to retrospectively outline the clinical and hemodynamic features derived non-invasively from echocardiography in patients with combined AS-MS to compare them with those of isolated severe AS. Additionally, the outcomes and prognostic indicators of patients with combined AS-MS are compared to that of patients with isolated AS.

## 2 Methods

### 2.1 Study population

We retrospectively identified patients with significant (i.e., reported as ≥moderate) AS using our echocardiography laboratory database to identify patients with coexistent significant mitral stenosis (i.e., reported as ≥moderate). This study was conducted at the King Faisal Specialist Hospital and Research Centre, a tertiary hospital located in Saudi Arabia between the years of 2003 to 2018. AS was defined as an aortic valve area less than or equal to 1.0 cm^2^ irrespective of gradient and flow (11, 12). Significant mitral stenosis was defined as a mitral valve area less than or equal to 2.0 cm^2^ irrespective of gradient (11, 12). Excluded patients included those with an LVEF < 50%, the coexistence of other significant valve lesions (i.e., ≥moderate mitral regurgitation and aortic regurgitation), patients who underwent prior cardiac surgery or, percutaneous mitral commissurotomy patients with associated congenital heart disease (e.g., aortic coarctation, atrial and/or ventricular septal defects), and patients with infective endocarditis. After exclusions, patients with isolated aortic stenosis were identified and compared with those presenting combined mitral and aortic stenosis. Aortic valve lesions included were either of rheumatic origin, bicuspid, or degenerative. All mitral lesions causing stenosis were of rheumatic origin.

This study was approved by the institutional review board at King Faisal Specialist Hospital & Research Centre under the approval number RAC 2251029.The requirement for written informed consent was waived by the ethics committee due to the retrospective nature of the study. All methods were carried out in accordance with relevant guidelines including the “Declaration of Helsinki”.

### 2.2 Data collection

All patients who met the inclusion criteria and had undergone appropriate investigations were retrospectively analyzed. Baseline clinical characteristics and outcome measures were collected from the patient's electronic medical records on admission. Demographic and clinical reported data included age, gender, height, weight, BMI, BSA, presence of persistent atrial ﬁbrillation, patients with a history of hypertension or those on current antihypertensive regimens, patients with diabetes mellitus, patients with a history of dyslipidemia or those on current anti hyperlipidemic agents, and patients with coronary artery disease (CAD). CAD was deﬁned by a previous history of CAD or any coronary artery stenosis >50% during coronary angiography or computed tomography scanning, or any imaging functional test positive for ischemia.

### 2.3 Transthoracic echocardiography

Transthoracic Echocardiography (TTE) was performed using the Vivid E95 by General Electric. Comprehensive TTE assessments including those of aortic and mitral stenosis were performed according to standard recommendations (13–15). AS was assessed utilizing a multiwindow approach, and the aortic valve area (AVA) was measured using the continuity equation (16). Significant AS was defined as an aortic valve area less than or equal to 1.0 cm2 or an indexed AVA less than or equal to 0.6 cm2 irrespective of gradient and flow (11, 12). Significant MS was defined as a mitral valve area less than or equal to 2.0 cm2 irrespective of gradient (11, 12). Aortic valve morphology and aortic valve stenosis severity using the peak and mean gradients were obtained using the Bernoulli equation (17). The mitral valve area (MVA) was measured both by planimetry using 2D echocardiography whenever echocardiography image quality allowed for it and the pressure half-time method in an apical four chamber view using continuous wave Doppler (13). Transmitral gradient (TMG) was obtained from the transmitral flow velocity waveform recorded by continuous wave Doppler. Chamber and LVEF quantification was based on standard recommendations (14). Left atrial diameter, interventricular septum thickness at end-diastole (IVSD), and LV posterior wall thickness in end-diastole (LVPWD) were measured in the parasternal long axis view. The dimensionless velocity index; the stroke volume (SV), and the indexed stroke volume to body surface area were also calculated according to the current guidelines (13–15). Systolic ejection time (ET) was measured using the left ventricular outflow tract velocity time integral Doppler. The corrected ejection time was calculated using the following formula: corrected ET = measured ET + heart rate ×1.7 for males or ×1.6 for females (18). The transvalvular flow rate (Q) was calculated using the following formula: Q flow = SV/ET (19). The tricuspid valve was also assessed for presence of stenosis and regurgitation or both. Peak transvalvular velocity was measured, and tricuspid regurgitation severity was assessed semi quantitatively and classified as mild, moderate or severe. Systolic pulmonary artery pressure was extrapolated using the peak TR velocity and the RA pressure. The RA pressure was evaluated using the inferior vena cava dimension and collapsibility as currently recommended. Rheumatic MS was defined by echocardiography when typical features such as leaflet thickening, nodularity, commissural fusion, and chordal fusion and shortening were present.

TTE was performed by multiple sonographers when clinically indicated and in accordance with standard clinical practice guidelines. In addition, echocardiographic parameters and images were independently reviewed by experienced level 3 trained echocardiographers.

### 2.4 Statistical analysis

Continuous data are expressed as mean ± standard deviations (SD) and are compared using t-test. Categorical data are summarized according to their frequency and percentages and compared with a chi-squared (*χ*^2^) or Fisher exact test, as appropriate. Univariate and multivariate linear regression was used to assess predictors of aortic flow. All variables significant in the univariate model were included in the multivariate analysis. Probabilities of survival were obtained by Kaplan–Meier estimates and unadjusted comparison among the two main groups was done using a 2-sided log-rank test. The endpoint of survival was all-cause mortality during follow-up i.e., overall survival (OS). Univariate and multivariate modelling of survival was done using the Cox proportional hazard model. All predictors significant in univariate survival analysis were included in the primary multivariate model except for tricuspid regurgitation velocity, which was available only for 227 patients. For sensitivity analysis, a secondary model was also built that included the tricuspid regurgitation velocity. Furthermore, gender was included into the model based on previous knowledge. A secondary sensitivity analysis was done using full inverse weight matching on main demographic covariates—age and gender. The weighted Cox proportional hazard model was used to evaluate survival in the matched sample. All statistical analyses were performed using R version 4.3.0 (The R Foundation for Statistical Computing, Vienna, Austria). For all statistical tests, a *P* < 0.05 was considered signiﬁcant.

## 3 Results

**Figure 1:**
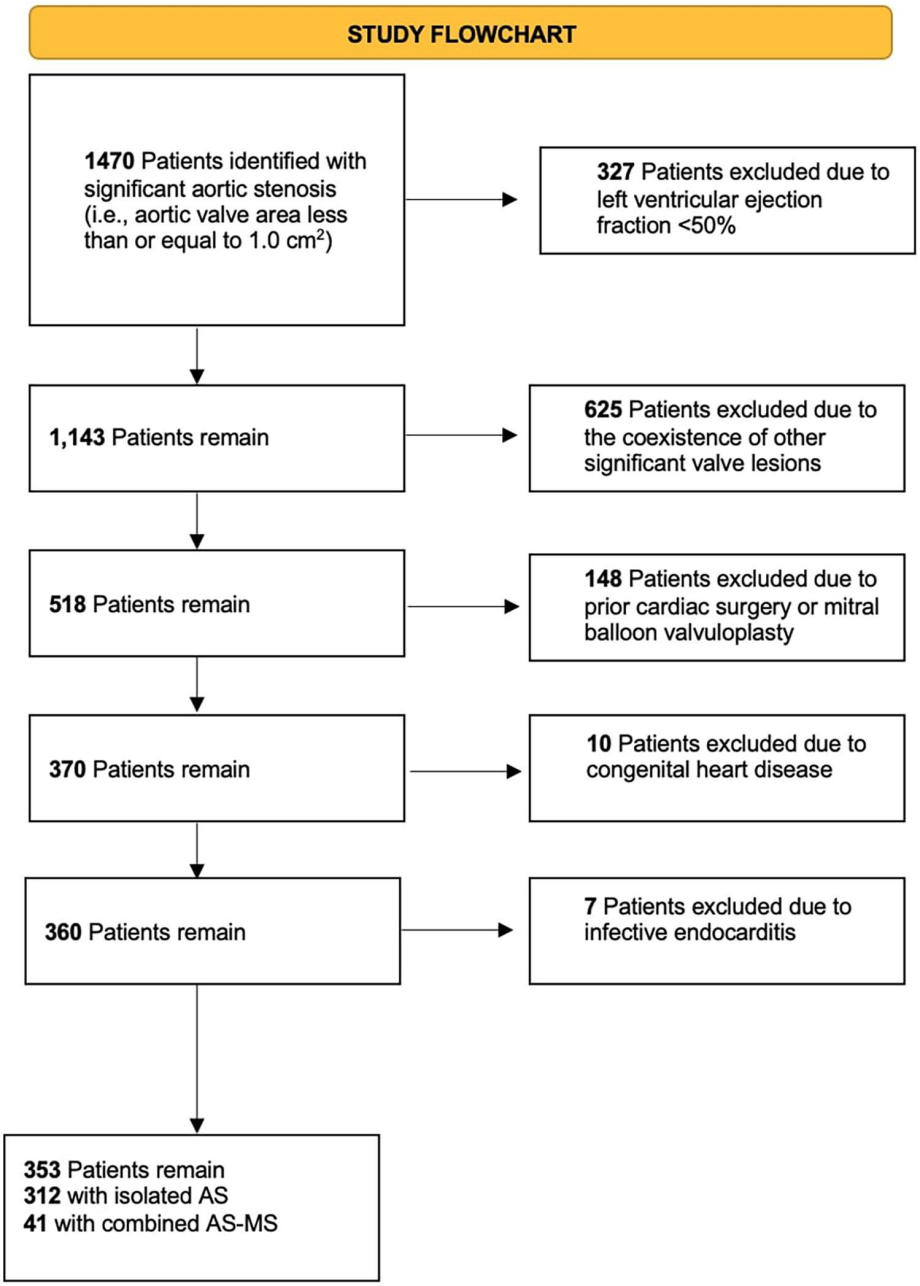
Study flowchart.

A total of 1,470 patients were identified as having significant AS (i.e., aortic valve area less than or equal to 1.0 cm2). Of these patients, 327 Patients were excluded due to having an LVEF < 50%. Additionally, 625 Patients were excluded due to the coexistence of other significant valve lesions (i.e., ≥ moderate mitral regurgitation and aortic regurgitation). Another 148 patients were excluded due to a history of prior cardiac surgery or percutaneous mitral commissurotomy. Finally, 10 patients were excluded due to a history of congenital heart disease whereas 7 patients were excluded due to infective endocarditis. In total, 353 patients were included in the study with 312 patients having isolated AS and 41 having combined AS-MS. Figure 1 summarizes the study flowchart.

### 3.1 Baseline characteristics

Table 1 summarizes the baseline characteristics of patients with combined AS-MS in comparison to those with isolated AS. The average age of patients in the isolated AS group was significantly higher than that of the combined AS-MS group (63 vs. 50 years, *p* < 0.001). Moreover, a higher proportion of patients in the isolated AS group had a history of hypertension compared to patients with combined AS-MS (64% vs. 44%, *p* = 0.017). Indeed, both the systolic blood pressure (132 mmHg vs. 116 mmHg, *p* < 0.001) and the diastolic blood pressure (71 mmHg vs. 67 mmHg, *p* = 0.02) were higher among patients with isolated AS compared to those with combined AS-MS. The prevalence of type 2 diabetes mellitus was observed in 42% of subjects with isolated AS compared to only 22% of subjects with combined AS-MS (*p* = 0.013). Interestingly, the prevalence of atrial fibrillation was significantly higher among patients with combined AS-MS (17%) compared to those with isolated AS (5%) (*p* = 0.003).

**Table 1:** Comparison of baseline clinical characteristics between patients with isolated AS and patients with combined AS-MS.

Characteristics	Whole cohort *n* = 353	Isolated AS *n* = 312	Combined AS-MS *n* = 41	*p* value
Age, years	61 ± 19	63 ± 18	50 ± 19	**<0**.**001**
Male gender, %	193 (55%)	175 (56%)	18 (44%)	0.14
Body surface area, m^2^	1.80 ± 0.23	1.81 ± 0.23	1.75 ± 0.23	0.13
History of hypertension, %	217 (61%)	200 (64%)	18 (44%)	**0**.**017**
Systolic blood pressure, mmHg	130 ± 21	132 ± 21	116 ± 18	**<0**.**001**
Diastolic blood pressure, mmHg	71 ± 12	71 ± 12	67 ± 12	**0**.**02**
Heart rate, beat/min	71 ± 13	71 ± 13	69 ± 14	0.38
Type 2 diabetes mellitus, %	141 (40%)	132 (42%)	9 (22%)	**0**.**013**
Dyslipidemia, %	125 (35%)	115 (37%)	10 (24%)	0.13
Atrial fibrillation, %	21 (6%)	14 (5%)	7 (17%)	**0**.**003**
Coronary artery disease, %	87 (25%)	82 (26%)	5 (12%)	0.06

Values are mean ± SD.

Bold values indicate statistical significance (*p* < 0.05).

AS, aortic stenosis; MS, mitral stenosis.

### 3.2 Echocardiographic parameters

The echocardiographic data including the comparison between the isolated AS and the combined AS-MS are shown in Table 2. All left ventricular dimensions including left ventricular mass and left ventricular ejection fraction were significantly different in both groups of patients. The prevalence of bicuspid aortic valves was 15% in the whole cohort and was significantly higher in the group of isolated AS compared to patients with combined AS-MS (17% vs. 2%, *p* = 0.015).

**Table 2:** Comparison of echocardiographic characteristics between patients with isolated AS and patients with combined AS-MS.

Characteristics	Whole cohort *n* = 353	Isolated AS *n* = 312	Combined AS-MS *n* = 41	*p* value
Aortic valve morphology				
Bicuspid aortic valve phenotype, %	54 (15%)	53 (17%)	1 (2%)	**0**.**015**
Flow-related data				
LV outflow tract diameter, cm	2.09 ± 0.23	2.09 ± 0.23	2.11 ± 0.23	0.63
LVOT VTI	22.4 ± 4.0	22.5 ± 4.0	21.7 ± 3.5	0.23
Stroke volume, ml	78 ± 21	78 ± 21	77 ± 21	0.78
Stroke volume index, ml/m^2^	43 ± 11	43 ± 11	45 ± 14	0.48
Stroke volume <35 ml/m^2^, %	83 (24%)	75 (24%)	8 (20%)	0.52
LVOT ejection time, ms	319 ± 36	319 ± 37	317 ± 33	0.75
Transvalvular flow rate, ml/s	245 ± 62	245 ± 61	244 ± 69	0.89
Cardiac output, L/min	5.44 ± 1.62	5.47 ± 1.60	5.26 ± 1.80	0.44
AS severity				
AV Peak velocity, m/s	4.25 ± 0.78	4.27 ± 0.78	4.11 ± 0.71	0.21
AV TVI	98.6 ± 22.6	98.9 ± 22.7	96.3 ± 22.1	0.49
Peak gradient, mm Hg	75 ± 28	76 ± 28	70 ± 23	0.17
Mean gradient, mm Hg	45 ± 17	46 ± 17	43 ± 15	0.31
Mean gradient ≥40 mm Hg, %	210 (60%)	188 (60%)	22 (54%)	0.42
AV area, cm^2^	0.82 ± 0.26	0.82 ± 0.26	0.84 ± 0.31	0.69
Indexed AV area, cm^2^/m^2^	0.46 ± 0.14	0.46 ± 0.14	0.48 ± 0.17	0.29
Doppler velocity index	0.24 ± 0.06	0.24 ± 0.06	0.24 ± 0.07	0.89
Cardiac chamber dimensions and function				
LV ejection fraction, %	61 ± 5	61 ± 5	60 ± 2	0.23
Interventricular septum, cm	1.07 ± 0.23	1.08 ± 0.22	1.03 ± 0.25	0.17
Posterior wall thickness, cm	0.99 ± 0.20	0.99 ± 0.21	0.97 ± 0.19	0.53
LV end-diastolic dimension, cm	4.55 ± 0.61	4.54 ± 0.62	4.56 ± 0.60	0.87
LV end-systolic dimension, cm	2.86 ± 0.51	2.85 ± 0.50	2.97 ± 0.60	0.15
LV mass, g/m²	93 ± 29	93 ± 29	92 ± 29	0.86
LV hypertrophy, %	89 (27%)	76 (26%)	13 (32%)	0.45
LA dimension, cm	4.05 ± 0.78	3.93 ± 0.73	4.79 ± 0.70	**<0**.**001**
Tricuspid valve				
Tricuspid peak gradient, mmHg	32 ± 11	30 ± 10	40 ± 15	**<0**.**001**
Tricuspid peak velocity, m/s	2.77 ± 0.49	2.71 ± 0.45	3.14 ± 0.59	**<0**.**001**
≥moderate tricuspid regurgitation	8 (2%)	2 (1%)	6 (15%)	**<0**.**001**
Mitral valve				
Mitral valve PHT, ms	–	–	168 ± 50	–
Mitral peak gradient, mm Hg	–	–	16.8 ± 7.0	–
Mitral mean gradient, mm Hg	–	–	9.5 ± 5.8	–
Mitral valve area (planimetry; *n* = 24), cm^2^	–	–	1.17 ± 0.38	–
Mitral valve area (PHT), cm^2^	–	–	1.36 ± 0.32	–

Values are mean ± SD.

Bold values indicate statistical significance (*p* < 0.05).

AS, aortic stenosis; LA, left atrium; LV, left ventricle; LVOT, left ventricular outflow track; MS, mitral stenosis; PHT, pressure half time; TVI, velocity-time integral; AV, aortic valve.

Echocardiographic parameters to assess AS severity did not show any significant differences between both groups in terms of aortic peak velocity, peak and mean gradients, aortic valve area, and Doppler velocity index. Moreover, corrected ejection time was significantly prolonged in the whole cohort and did not differ between both groups, suggesting truly severe AS. The percentage of patients with paradoxical low mean pressure gradient <40 mmHg despite preserved ejection was not higher in the group of concomitant AS-MS compared to the isolated AS group (46% vs. 40%, *p* = 0.42).

As expected, patients with associated MS had all classical echocardiographic criteria of MS severity including peak and mean gradients, pressure half time and mitral valve area either measured by planimetry (1.17 ± 0.38 cm^2^) or by the pressure half time formula (1.36 ± 0.32 cm^2^). The left atrium was significantly larger in the AS-MS patient group (4.79 ± 0.70 cm vs. 3.93 ± 0.73 cm, *p* < 0.001). Additionally, the peak tricuspid velocity was higher in the AS-MS group than in the isolated AS group (3.14 ± 0.59 m/s vs. 2.72 ± 0.45 m/s, *p* < 0.001) suggestive of a more advanced degree of increased right ventricular systolic pressure. Furthermore, a significantly higher proportion of patients with combined AS-MS had moderate or severe tricuspid regurgitation (15%) compared to those with isolated AS (1%) (*p* < 0.001).

**Figure 2:**
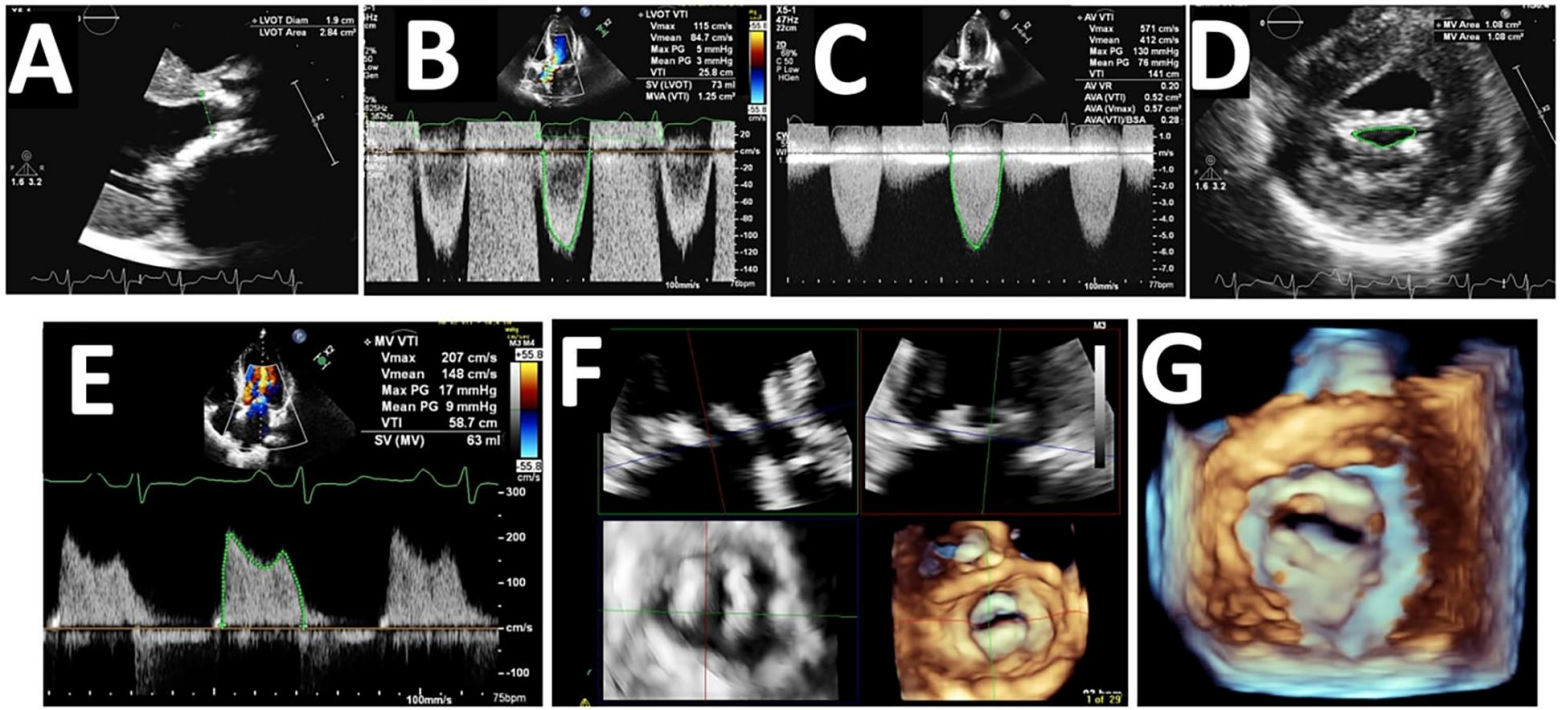
Echocardiographic findings in a patient with concomitant aortic stenosis and mitral stenosis. **(A)** Parasternal long-axis view showing the left ventricular outflow tract (LVOT) diameter (1.9 cm) and area (2.84 cm^2^); **(B)** Pulse-wave Doppler tracing at the LVOT showing velocity-time integral (VTI) of 25.8 cm and calculated stroke volume (SV) of 73 ml, with a mitral valve area (MVA) of 1.25 cm^2^ derived from VTI; **(C)** Continuous-wave Doppler tracing across the aortic valve showing a peak velocity of 571 cm/s and mean gradient of 76 mmHg, with a calculated aortic valve area of 0.57 cm^2^, indicating severe AS; **(D)** 2D parasternal short-axis view of the mitral valve showing MVA by planimetry (1.08 cm^2^), consistent with severe MS; **(E)** Continuous-wave Doppler of the mitral valve showing peak velocity of 207 cm/s and mean transmitral gradient of 9 mmHg, further confirming MS severity; **(F)** 3D echocardiographic multiplanar reconstruction of the mitral valve, allowing detailed anatomical evaluation of leaflet fusion and valve morphology; **(G)**: 3D-rendered image of the mitral valve orifice, showing the restricted valve opening and commissural fusion in severe MS.

The stroke volume index was 43 ± 11 ml/m^2^ in the whole cohort without significant differences between the two groups. Moreover, the percentage of those with “paradoxical” low stroke indexed < 35 ml/m^2^ was 24% in the whole cohort and was not higher in the group with associated MS (*p* = 0.52). Lastly, no difference was observed in the transvalvular flow rate between the two groups (*p* = 0.89). A representative echocardiographic case of concomitant AS-MS is shown in Figure 2.

### 3.3 Determinants of transvalvular flow rate/paradoxical low-flow, low-gradient as

By univariate analysis, the factors associated with flow rate are shown in Table 3. Age, gender, body surface area, presence of hypertension, diabetes, dyslipidemia, and CAD were associated with flow rate (all *p* values < 0.05). Additionally, aortic valve area, bicuspid morphology, ejection fraction, and left ventricular end diastolic dimensions were univariate determinants of flow rate. In multivariable analysis, body surface area and aortic valve area remained significant predictors of flow rate.

**Table 3:** Univariate and multivariate analysis of the predictors of transvalvular flow rate.

Characteristics	Univariate		Multivariate	
	Coefficient ± SE	*p* value	Coefficient ± SE	*p* value
Age, years	**−0.6** **±** **0.2**	**0**.**0007**	−0.001 ± 0.15	0.9945
Male gender	**−20.6** **±** **6.6**	**0**.**0018**	0.4 ± 4.5	0.9224
Body surface area (m^2^)	**85.6** **±** **13.7**	**<0**.**0001**	**33.5** **±** **10.2**	**0**.**0011**
Hypertension	**−19.5** **±** **6.7**	**0**.**0039**	−7.8 ± 5.4	0.1503
Systolic blood pressure, mm Hg	−0.20 ± 0.2	0.187	–	–
Diabetes	**−13.7** **±** **6.7**	**0**.**0428**	−9.6 ± 5.1	0.0621
Dyslipidemia	**−13.9** **±** **7.0**	**0**.**0445**	−0.6 ± 6.6	0.9298
Atrial fibrillation	−10.0 ± 14.0	0.474	–	–
Coronary artery disease	**−16.6** **±** **7.6**	**0**.**0308**	−5.2 ± 7.1	0.4632
Peak aortic gradient, mmHg	−0.1 ± 0.1	0.356	–	–
Aortic valve area, cm^2^	**183.0** **±** **8.1**	**<0**.**0001**	**172.2** **±** **8.1**	**<0**.**0001**
Bicuspid aortic valve	**33.3** **±** **9.0**	**<0**.**0003**	11.8 ± 6.6	0.0763
LV ejection fraction, %	**1.6** **±** **0.6**	**0**.**0153**	0.7 ± 0.4	0.0737
LV end-diastolic dimension, cm	**2.0** **±** **0.5**	**0**.**0003**	0.5 ± 0.4	0.1723
LV mass index, g.m^−2.7^	0.02 ± 0.12	0.859	–	–
Tricuspid peak velocity, m/s	−0.008 ± 0.082	0.924	–	–
≥moderate TR	−20.9 ± 22.2	0.345	–	–
Presence of concomitant MS	**−**1.4 ± 10.3	0.893	–	–

Bold values indicate statistical significance (*p* < 0.05).

LV, left ventricle; TR, tricuspid regurgitation; SE, standard error.

We also analyzed differences among groups in prediction of transvalvular flow. There were significant interactions for presence of arterial hypertension (−25.4 ± 7.1 for AS group but 18.6 ± 21.7 for AS-MS group, *p* for interaction = 0.03) and dyslipidemia (−19.4 ± 7.1 for AS group but 36.7 ± 24.6 for AS-MS group, p for interaction = 0.018).

### 3.4 Outcome measures

**Figure 3:**
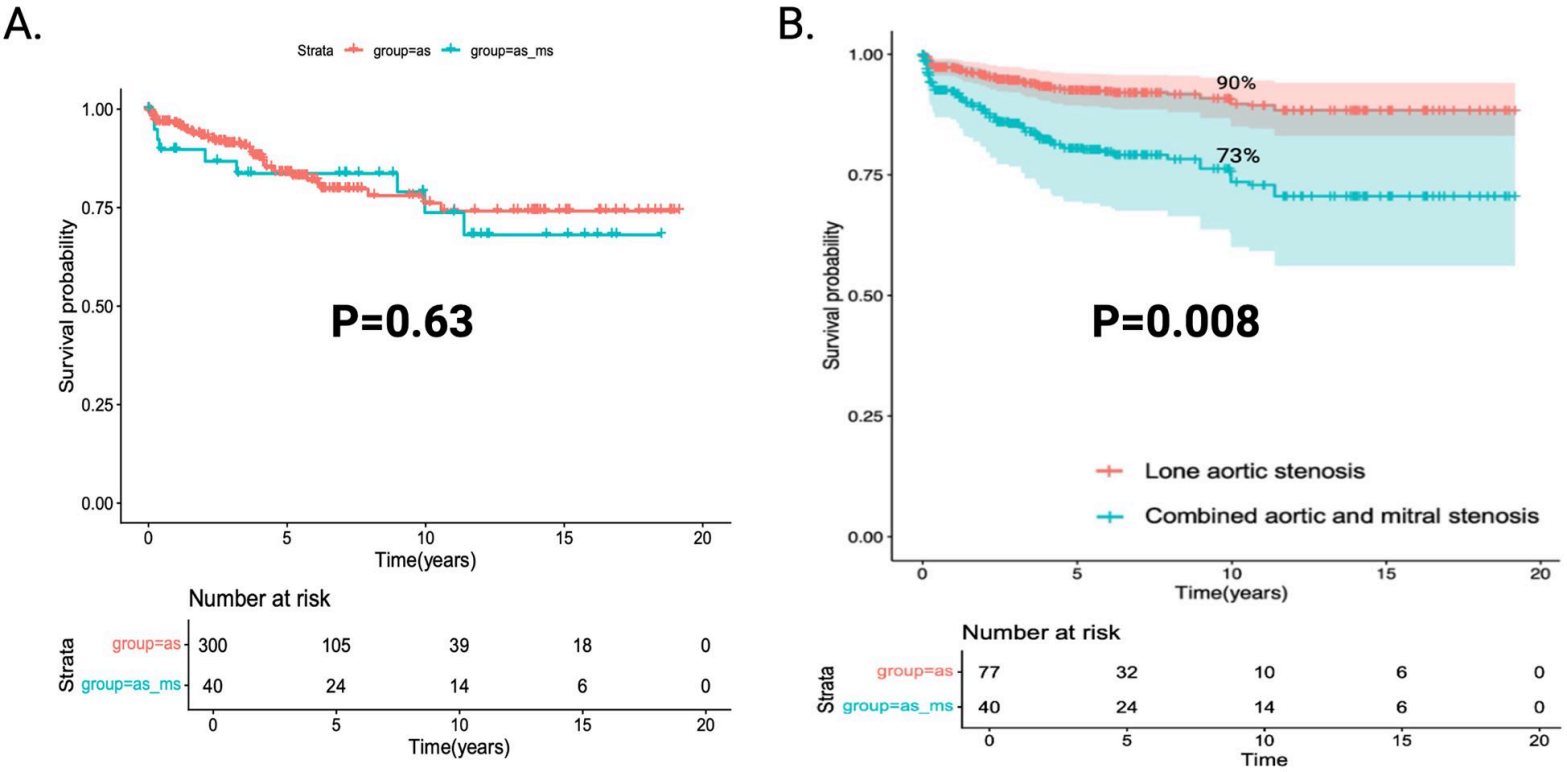
Kaplan–Meier curve comparing both the unadjusted and the predicted matched 5-year overall survival rate between the isolated aS group and the combined aS-MS group. **(A)** 5-year unadjusted overall survival rate as compared between the isolated AS group and the combined AS-MS group. **(B)** Weighted cox proportional hazards model predictions using inverse probability weight matching comparing the isolated AS group and the combined AS-MS group using matching for gender and age.

Overall, there were 48 deaths in the whole cohort. Mean follow-up time was 5.02 years [range 0.2 years to 19 years]. The 5-year overall survival rate of the whole cohort was 84% [95% CI 79%–89%]. The unadjusted survival rate in the isolated AS group was 84% [95% CI 79%–89%] at 5 years, which was the same as that in the combined AS-MS group [84% (95% CI 72%–97%)]. Overall, there were no differences in the unadjusted 5-year overall survival rate (Figure 3A, *p* = 0.63).

Table 4 demonstrates the predictors of overall survival by univariate and multivariable analysis. By univariate analysis, concomitant AS-MS was not significantly associated with overall mortality (HR = 1.2, 95% CI 0.576–2.5 *p* = 0.626). Age and the presence of comorbidities (diabetes, hypertension, atrial fibrillation) was significantly associated with worsened overall survival. Furthermore, among echocardiographic parameters, LV dimension, mean aortic gradient, and tricuspid regurgitation velocity were associated with outcome. In addition, the presence of moderate or severe tricuspid regurgitation was significantly associated with poorer overall survival [HR 2.58 (95% CI 1.16–5.77)].

**Table 4:** Univariate and multivariate analysis of the predictors of overall survival.

Characteristics	Univariate		Multivariate	
	HR [95CI]	*p* value	HR [95CI]	*p* value
AS-MS group	1.2 [0.576–2.5]	0.626	**2.672 [1.060–6.732]**	**0**.**03713**
Age, years	**1.04 [1.02–1.05]**	**0**.**000118**	**1.028 [1.004–1.053]**	**0**.**02192**
Male gender	1.5 [0.853–2.66]	0.159	1.446 [0.742–2.819]	0.27876
Body surface area (m^2^)	1.18 [0.334–4.17]	0.796	–	–
Hypertension	**2.53 [1.29–4.97]**	**0**.**00711**	1.100 [0.441–2.744]	0.83783
Heart rate	**1.03 [1.01–1.05]**	**0**.**00109**	**1.026 [1.005–1.048]**	**0**.**01587**
Systolic blood pressure, mm Hg	0.995 [0.981–1.01]	0.442	–	–
Diastolic blood pressure, mm Hg	0.98 [0.957–1]	0.0975	–	–
Diabetes	**1.97 [1.11–3.47]**	**0**.**02**	0.881 [0.431–1.799]	0.72761
Dyslipidemia	1.1 [0.607–1.98]	0.759	–	–
Atrial fibrillation	**2.58 [1.16–5.77]**	**0**.**0205**	2.054 [0.827–5.103]	0.12096
Coronary artery disease	1.6 [0.866–2.94]	0.134	–	–
Peak aortic gradient, mmHg	0.988 [0.977–1]	0.0521	–	–
Mean aortic gradient, mmHg	**0.973 [0.954–0.993]**	**0**.**00843**	**0.971 [0.950–0.992]**	**0**.**00786**
Aortic valve area, cm^2^	1.05 [0.349–3.19]	0.925	–	–
Bicuspid aortic valve	0.622 [0.264–1.46]	0.277	–	–
LV ejection fraction, %	0.981 [0.922–1.04]	0.55	–	–
LV end-diastolic dimension, cm	**0.888 [0.844–0.935]**	**<0.0001**	**0.912 [0.842–0.986]**	**0**.**02145**
Stroke volume index (ml/m^2^)	**0.955 [0.926–0.985]**	**0**.**0035**	**0.967 [0.935–1.000]**	**0**.**04965**
LV end-systolic dimension, cm	**0.911 [0.857–0.969]**	**0**.**0032**	0.985 [0.894–1.084]	0.75448
LV mass index, g.m^−2.7^	0.99 [0.978–1]	0.0841	–	–
Tricuspid peak velocity, m/s (*n* = 227)	**1.01 [1.0004–1.01]**	**0**.**0359**	–	–
≥moderate TR	**2.58 [1.16–5.77]**	**0**.**0205**	0.630 [0.114–3.487]	0.59692

Bold values indicate statistical significance (*p* < 0.05).

AS-MS, combinded aortic and mitral stenosis; LV, left ventricle; TR, tricuspid regurgitation; HR, hazard ratio; 95CI, 95 percent confidence intervals.

After multivariate adjustment, AS-MS was associated with worsened survival when compared to the isolated AS group with a hazard ratio of 2.672 [(95% CI 1.060–6.732), *p* = 0.037]. This remained significant even when using a secondary adjustment model that included tricuspid regurgitation velocity [HR 3.851 (1.2717–11.66), *p* = 0.0171]. Using inverse weight matching with age and gender as a secondary sensitivity analysis, a similar result for the AS-MS group was reached [HR 2.828 (1.312–6.096), *p* = 0.008]. Adjusted predicted survival curves based on the matching are shown in Figure 3B.

## 4 Discussion

We conducted a single-center retrospective study to evaluate the clinical characteristics, hemodynamic and echocardiographic features, and outcomes of patients with combined significant rheumatic MS and AS. The pertinent findings of our study are the following: (i) In this large cohort derived from a population where rheumatic valve disease is highly prevalent, the prevalence of significant concomitant MS and AS remains somewhat common with a prevalence of approximately 11%; (ii) The presence of concomitant MS does not appear to cause a higher rate of paradoxical low flow low gradient AS as previously reported; and (iii) After adjusting for age and gender the 5-year overall survival of combined AS-MS appears to be significantly lower than that of isolated AS.

The prevalence rate of 11% for combined AS-MS suggests that this association is somewhat prevalent in clinical practice. A limited number of studies have explored the prevalence of combined AS-MS. The combination of AS and MS has been previously reported in 17% of 170 patients undergoing combined mitral and aortic surgery in Switzerland (5). More recently, Joseph et al. utilized a large U.S. transcatheter valve therapies registry to investigate the prevalence of MS in patients undergoing transcatheter aortic valve replacement (TAVR) (6). The study found that the prevalence of MS was 11.6% in this cohort, with 2.7% of patients having severe disease. However, these studies were conducted in western countries where the prevalence of rheumatic valve disease is exceedingly low, making calcific MS the most probable etiology. Indeed, this is apparent when comparing the age of participants in the aforementioned studies, with the study conducted in Switzerland reporting a mean age of 82 years for patients with combined AS-MS, whereas the U.S. study reported a mean age of 76 for patients with combined AS-MS (5, 6). Within our study, we found that patients with combined AS-MS were significantly younger (mean age 50 years vs. 63 years, *P* < 0.001) with lower prevalence of comorbidities such as hypertension and diabetes. This is indicative of a younger population of patients being affected by rheumatic valve disease when compared to older patients with calcific aortic/mitral stenosis.

According to 2025 ESC/EACTS and 2020 ACC/AHA guidelines, severe AS is underpinned by a peak aortic jet velocity >4.0 m/s, a mean gradient >40 mmHg, or an aortic valve area (AVA) <1.0 cm^2^ (11, 12). However, discordance between the aortic valve area and the mean gradient occurs in approximately 20%–30% of cases which can consequently complicate the grading and management of AS (20). In patients with a preserved ejection fraction as included in our study, severe AS can be divided into four main categories in relation to flow (stroke volume index <35 or ≥35 mL/m^2^) and gradient (<40 or ≥40 mmHg): (i) normal-flow, high-gradient, (ii) normal-flow, low-gradient, (iii) low-flow, high-gradient, and (iv) low-flow, low-gradient (21). Low-flow, low-gradient (LF/LG) severe AS, occurring in the setting of a preserved ejection fraction is termed ‘paradoxical LF/LG’ as opposed to the classical subtype seen with a reduced ejection fraction (22, 23). The concomitance of severe AS and MS has frequently been reported to be a cause of paradoxical LF/LG (7–9, 23). Theoretically, the presence of concomitant MS should impair preload and reduce forward stroke volume resulting in a reduction in the transvalvular flow rate and the onset of paradoxical LF/LG AS (9, 23). However, within our study both the gradient and the transvalvular flow rate did not differ between the combined AS-MS group and the isolated AS group. Moreover, the presence of combined AS-MS was not a significant predictor of transvalvular flow rate at both univariate and multivariate analyses. Therefore, our study suggests that the presence of concomitant rheumatic MS and significant AS does not tend to cause a higher prevalence of paradoxical LF/LG AS as previously reported. One possible explanation for our findings is that there are compensatory mechanisms which increase stroke volume in the setting of combined AS-MS.

The presence of concomitant severe AS-MS raises several important diagnostic implications. Indeed, the coexistence of both diseases makes their hemodynamic assessment particularly difficult due to interactions between both disorders which hinder the diagnostic accuracy of specific methods which are largely validated in the setting of isolated disease. For instance, the pressure-half-time method may be inaccurate in evaluating the mitral valve area in the setting of severe AS due to the impaired left ventricular diastolic function which can consequently lead to an overestimation of the mitral valve area (24, 25). Additionally, the continuity equation for calculating the aortic valve area and mitral valve area may be inaccurate in this as it can result in the overestimation of MS in the setting of severe AS (9, 26). Therefore, in the setting of concomitant AS-MS where MS is rheumatic in origin and calcification is minimal, 2D and 3D planimetry is critical. Additionally, Dobutamine stress echo, transesophageal echocardiography and/or cardiac catheterization may be necessary in situations of diagnostic uncertainty.

The outcomes of patients with combined AS-MS in our study were comparable to those of isolated AS at first glance. However, we hypothesized that this is likely mediated by the lower age of patients affected by rheumatic MS when compared to isolated AS which is of degenerative etiology in a majority of patients. Therefore, after adjusting for age and gender, the outcomes of patients with combined AS-MS appeared to be significantly worse than that of those with isolated AS. Additionally, the presence of combined AS-MS was an independent predictor of mortality at multivariate analysis. This is in concordance with other studies evaluating the outcome of combined AS-MS (9, 27, 28). This finding has important clinical implications as it is imperative to adequately assess patients with concomitant rheumatic MS and AS without delaying valve interventions.

Our study has several limitations. Firstly, the retrospective nature of our study is subject to inherent limitations including potential missing data and incomplete medical records. Moreover, the single-center nature of our study limits the sample size and demographic distribution of our study, hindering its applicability to a general population. Additionally, the small sample size of our study limits the power of our study and increases the likelihood of a type 2 error. Moreover, given the retrospective nature of this study, detailed mitral valve parameters were not systematically assessed in patients with isolated AS once MS was excluded on 2D echocardiography, and planimetry was not performed. We acknowledge this limitation and emphasize that future prospective studies should incorporate systematic mitral valve assessment even in isolated AS patients, as subtle mitral involvement may influence flow dynamics and outcomes. The measurement of aortic valve area by the continuity equation and the mitral valve area by the pressure-half-time method in the setting of combined AS-MS can lead to inaccurate estimation of these parameters. This study relied on non-invasive TTE undertaken as clinically indicated; however, the utilization of other imaging modalities (stress echocardiography, TEE, CT, or cardiac catheterization) may have had additional diagnostic value in assessing the hemodynamics of combined AS-MS. In particular, our analysis was based solely on resting echocardiographic data, which may not fully capture the hemodynamic complexity of patients with combined AS and MS. We did not assess dynamic changes in cardiac output or transmitral flow that occur during exertion or stress, where diastolic filling time and left atrial pressure play a critical role. This limitation is especially relevant in the setting of coexisting severe AS, where the capacity to augment cardiac output during physical activity may be impaired. As a result, symptom burden or functional limitation may have been underestimated when assessed only at rest. Future studies incorporating stress echocardiography or invasive exercise hemodynamic measurements would provide a more comprehensive understanding of these interactions. Lastly, the higher prevalence of bicuspid aortic valve (BAV) among those with isolated AS as opposed to those with combined AS-MS may be due to TTE having a poor sensitivity for BAV diagnosis (as opposed to CT), and this poor sensitivity might be even lower among patients with RHD (29). This further highlights the importance of multimodality imaging in patients with MVHD.

## 5 Conclusions

In conclusion, in this large cohort derived from a population where rheumatic valve disease is highly prevalent, the prevalence of significant concomitant mitral stenosis and aortic stenosis remains somewhat common at approximately 11%. Additionally, the presence of concomitant mitral stenosis does not appear to cause a higher rate of paradoxical low flow low gradient aortic stenosis as previously reported. Lastly, the outcome of patients with combined AS-MS appears to be significantly worse than that of isolated AS. Larger global studies are required to explore the true prevalence of combined AS-MS in prospective based-population studies. Moreover, future studies are required to confirm our finding that the presence of MS does not increase the prevalence of paradoxical low-flow, low-gradient AS. In addition, studies should explore the hemodynamic and physiological factors which cause the maintenance of flow and gradient in the setting of combined AS-MS. Furthermore, more evidence is needed to validate diagnostic methodologies in the setting of combined AS-MS.

## Data Availability

The raw data supporting the conclusions of this article will be made available by the authors, without undue reservation.
